# Supplementary material for: The added value of cognition-targeted exercise versus symptom-targeted exercise for multiple sclerosis fatigue: A randomized controlled pilot trial
Source: PLoS One. 2021 Nov 8;16(11):e0258752. doi: 10.1371/journal.pone.0258752 (PMC8575272; doi:10.1371/journal.pone.0258752)
Supplement: S1 File — (PDF) [file pone.0258752.s002.pdf]

Research Ethics Committee

Cairo University

Application for Approval of a Project Involving Human Subjects

Table of Contents

1- Title “ 3

2- Introduction 3

3- METHODS 4

4. Risks 9

5. Recruitment 10

6- Allocation 10

1- APPLICATION DETAILS

1.1- Project title **"The Added Value of Cognition-targeted Exercise Versus Symptom-targeted Exercise for Multiple Sclerosis Fatigue: Randomized Controlled Trial**

.

Arabic title:

القيمة المضافة للتمرين الموجه للإدراك مقابل التمرين الموجه للأعراض لإرهاق التصلب المتعدد: تجربة عشوائية خاضعة للرقابة

1.2 Project funding and duration

What is the proposed starting date? January 1, 2021
What is the expected duration of this research 6 months

1.3 Applicants' details

Name of the Principle Investigator: Ibrahim Moustafa
(In case of undergraduate student research projects, the principle investigator will be the supervising faculty member)
Academic rank (applicable to faculty members): Associate professor
Mobile Tel.: 00971502180024 Email: __Ibrahiem. mostafa@ pt.cu.edu.eg

2- Introduction

2.1Background and rational

Whilst the role of Cognitive behaviour therapy (CBT) in reducing fatigue severity is well established [1][2][3],there are a lot of controversies regarding the role that physical activities and exercises play in CBT. Several studies support that the effect of CBT has often been attributed to an increase in activity because physical exercises have traditionally played a major role in CBT.

[4] [5] [6][7].In contrast , a substantial body of research is also available to support that the effect of CBT on fatigue is not mediated by a persistent increase in physical activity.[8][9][1]

In the light of these findings, we can not find a clear answer to the question whether the different types of exercise may affect the fatigue differently.Taking these considerations into account, the purpose of the present study was to determine whether the treatment effect of CBT on fatigue is mediated by adding different exercise approach (time-contingent exercises versus symptom-contingent exercises) .Such a finding would be important to the validation of all treatment approaches which rely on CBP along with physical exercise programs in some way to improve fatigue.

2.2 **Objectives**

The objective of this study was to explore the added value effect of cognition-targeted exercise versus symptom-targeted exercise for MS fatigue.

2.3 **Trial design**

Randomized controlled trial with 1 month follow up

2.4 **Please explain how your selected design relates, or is appropriate, to Fulfilling your objectives?**

The RCT is the most scientifically rigorous method of hypothesis testing available, and is regarded as the gold standard trial for evaluating the effectiveness of interventions. In the current study we will be concerned mainly with causal relationship, in addition, this true experimental design will allow us to control the threats for the internal validity of the current study .

3- METHODS

3.**1Study setting**

Research laboratory of Cairo university

3.2 **Eligibility criteria**

Participants will be included if they will be diagnosed with multiple sclerosis by a neurologist. • Being within normal or average dysfunction and excluding those scoring ≤6 in the Expanded Disability Status Scale (EDSS); • Being identified as a case level of fatigue; fatigue score of 4 or greater on the Fatigue Scale (FS);

Exclusion criteria will include patients with any serious psychological disorders (including psychotic disorders or active substance abuse), or those with any chronic illness that may be contributing to fatigue were excluded.

3.3 **Explain your sampling method if you are going to be using one.**

The researcher needs to describe clearly how the sample will be selected.

Our sample is a purposive sample (also known as judgment, selective or subjective sampling). This is a sampling technique in which researcher relies on his or her own judgment when choosing members of population to participate in the study.

3.4 Interventions

Experimental group : cognition-targeted exercise The patients in this group will receive cognitive behavior therapy in addition to cognition-targeted exercise

Control group. The patients in this group will receive cognitive behavior therapy in addition to symptom-targeted exercise .

cognition-targeted exercise All Standardized physical therapy exercises will be performed in a time-contingent . Goal setting is essentially done together with the patient, focussing on functionality instead of fatigue relief. Progression to a next level of (more difficult) exercises will be preceded by an intermediate phase of motor imagery. The treating physical therapist will be advised to address patients' cognitions about their problems during the cognition-targeted exercise

training, so that patients will have positive perceptions regarding their illness and treatment outcome. The treating physical therapist will be advised to discuss the patient's perceptions about each exercise. This include discussion of the anticipated consequences of the exercises and challenging the patient's cognitions in relation to the exercises Behavioral: Cognitive behavioral therapy In the first and second sessions, patients will learn about fatigue-related symptoms as well as Cognitive behavioral therapy and its effectiveness. The third and fourth sessions will be devoted on the introduction of behavioral strategies. The fifth session will introduce cognitive strategies to decrease fatigue. The last three sessions will be about how to adopt the proposed strategies consistently.

Behavioral: Cognitive behavioral therapy In the first and second sessions, patients will learn about fatigue-related symptoms as well as Cognitive behavioral therapy and its effectiveness. The third and fourth sessions will be devoted on the introduction of behavioral strategies. The fifth session will introduce cognitive strategies to decrease fatigue. The last three sessions will be about how to adopt the proposed strategies consistently. Standardized physical therapy The standardized physical therapy will consist of eight half-hour individualized face to face physiotherapy sessions, over a 4-week period. this program will consist of twice#weekly supervised general aerobic, strengthening and flexibility exercise sessions .This exercise program is reflective of the general exercises typically undertaken within routine clinical practice

3.5 Outcomes

A series of outcome measures will be obtained at three intervals: (1) baseline, (2 at 4-weeks and, (3) after 3 months . primary outcome measure will include :

Change in Modified Fatigue Impact Scale The Modified Fatigue Impact Scale is a modified form of the Fatigue Impact Scale based on items derived from interviews with MS patients concerning how fatigue impacts their lives. Items on the Modified Fatigue Impact Scale are aggregated into three subscales (physical, cognitive, and psychosocial), as well as into a total Modified Fatigue Impact Scale score. All items are scaled so that higher scores indicate a greater impact of fatigue on a person’s activities.

Secondary outcome measures will include

Change in Work and Social Adjustment Scale The Work and Social Adjustment Scale , which assessed fatigue-related impairment in relation to a number of areas of the person's life. The maximum score is 40 .lower score is better

Change in Hospital Anxiety and Depression Scale The Hospital Anxiety and Depression Scale , a commonly used self-report instrument for detecting states of depression and anxiety in patients with medical illnesses. scores of less than 7 indicate non-cases and high scores indicate more anxiety and depression

Change in Perceived Stress Scale the Perceived Stress Scale will be included to assess whether the degree to which patients appraised situations in their lives as stressful changed after the intervention. Individual scores on the Perceived Stress Scale can range from 0 to 40 with higher scores indicating higher perceived stress.

3.6 Sample size

The effect size estimate regarding the primary outcome MFIS , was obtained from a pilot study in which 10 participants underwent a similar protocol.. The effect size for the differences between the groups was estimated to be 0.79. Accordingly, given a significance level of 5% and statistical power of 80%,, a 25 patients for each treatment arm were needed . To compensate for potential attrition, we will increase the sample size by 20%.giving a total number of 30 patients per group.

3.7 **Are you in any relation with the research participants, which can influence Their voluntary consent?**

Such as recruiting students by faculty, or recruiting patients by their treating doctor.

No

3.8 Are you requesting a waiver of consent?

Information sheet should always be presented to research participants. In rare cases, the signed informed consent can be waived.

No

3.9 How will you guarantee understandability of the information which will be Presented to the research participants?

(As a general rule, the information in the informed consent must be in the participants' native Language and must be written in simple and understandable terms.)

Consent document are clearly written and understandable to subjects. The language is non-technical .Furthermore, consent forms will be provided in the language of the subject (Arabic or English versions). After explaining the research procedure to the participants, we will ask them to explain to us what they understood of it and answer any posed questions that they may have.

3.10 Research participants should have the right to withdraw at any time from The research. Explain what will you do if a participant decided to withdraw From your research?

For example, how will the already obtained data or samples be managed?

In the current study we will select Intent-to-treat analysis approach which is the gold standard for randomized clinical trials . In ITT analysis, data from all subjects initially enrolled in a clinical trial are used for the analysis and we will impute any missed data through multiple regression model. Of interest, to account for possible participant drop-outs, the sample size will increase by 10%.

3.11Please explain how will you ensure the privacy of the research setting?

This includes the invitation to participate in research, and the conducting of the research itself. For Example, examination will be conducted in a closed room…etc.
Write N/A if this is not applicable.

Physical assessment and treatment will be conducted in a closed and secured room. Assessment and treatment will be conducted by the same gender therapist .

We will avoid retaining for any information obtained at screening without the consent of those who either failed to qualify or refused to participate for possible future studies participation.

3.12 What steps have been taken to preserve confidentiality?

While it is impossible to collect data anonymously, the identifiers will be removed and destroyed immediately after the end of the trial additionally we will assign security codes to computerized records and securely store data documents within lock locations in the principal investigators office.

3.13 Where will the data (or samples) be stored?

It will be stored in a password encrypted file on the laptop of the principal investigators which will always be locked and password protected. Only the researchers will have access to the data.

4. Risks

4.1 Does this study involve any physical risks to participants?

Yes

Physical risks may include mild physical discomfort due to exercise.

4.2 **Does this study involve any psychological risks to participants?**

No

4.3 Does the study involve any social risk to participants?

No

4.4 Does the study involve any risk on the community's values?

No

4.5 Does this study require participants to release information of sensitive or

Personal nature?

No

4.6 Are there any other risks different from those encountered in everyday?

life?

No

4.7 What steps have you taken to minimize any of the above risks if applicable?

Write N/A if not applicable.
This risk can be minimized by closely monitor the patients during exercise program

4.8 Describe the circumstances under which the study could be stopped early.

Write N/A if not applicable.
If we found evidence of no important difference between experimental and control interventions.

5. - Allocation

Participants will be randomly assigned to an intervention group (n=40) or control group (n=40) according to a random number generator and restricted to permuted blocks of different sizes, with the researcher blinded to the sequence designated for each person

6- Data analysis

Variance homogeneity will be tested with Levene's test, obtaining a 95% confidence level and p-value > .05 and confirming variance equality. Descriptive statistics (means ± SD unless otherwise stated) will summarized at each time point. Student's t-test for continuous variables or chi-squared for categorical variables will be performed.

We will use an intention-to-treat approach with alpha set at .05 level of significance for all analyses. Comparative treatment effects of the two alternative treatments over the course of the 3-month follow-up will examined with 2-way analysis of variance (ANOVA) with repeated measures.

References

[1] J.F. Wiborg, H. Knoop, M. Stulemeijer, J.B. Prins, G. Bleijenberg, How does cognitive behaviour therapy reduce fatigue in patients with chronic fatigue syndrome? the role of physical activity, Psychol. Med. 40 (2010) 1281–1287. https://doi.org/10.1017/S0033291709992212.

[2] J.M. Malouff, E.B. Thorsteinsson, S.E. Rooke, N. Bhullar, N.S. Schutte, Efficacy of cognitive behavioral therapy for chronic fatigue syndrome: A meta-analysis, Clin. Psychol. Rev. 28 (2008) 736–745. https://doi.org/10.1016/j.cpr.2007.10.004.

[3] J.R. Price, E. Mitchell, E. Tidy, V. Hunot, Cognitive behaviour therapy for chronic fatigue syndrome in adults, Cochrane Database Syst. Rev. 2008 (2008). https://doi.org/10.1002/14651858.CD001027.pub2.

[4] A.J. Wearden, R.K. Morriss, R. Mullis, P.L. Strickland, D.J. Pearson, L. Appleby, I.T. Campbell, J.A. Morris, Randomised, double-blind, placebo-controlled treatment trial of fluoxetine and graded exercise for chronic fatigue syndrome, Br. J. Psychiatry. 172 (1998) 485–492. https://doi.org/10.1192/bjp.172.6.485.

[5] M. Sharpe, K. Hawton, S. Simkin, C. Surawy, A. Hackmann, I. Klimes, T. Peto, D. Warrell, S. Seagroatt, A. Nanke, Cognitive behaviour therapy for the chronic fatigue syndrome: A randomised controlled trial, Verhaltenstherapie. 8 (1998) 118–124. https://doi.org/10.1159/000030637.

[6] Cognitive Behavioural Therapy for Chronic Fatigue Syndrome: A Guide fo, (n.d.). https://www.routledge.com/Cognitive-Behavioural-Therapy-for-Chronic-Fatigue-Syndrome-A-Guide-for/Kinsella/p/book/9780415436120 (accessed April 17, 2021).

[7] L. Quarmby, K.A. Rimes, A. Deale, S. Wessely, T. Chalder, Cognitive-behaviour therapy for chronic fatigue syndrome: Comparison of outcomes within and outside the confines of a randomised controlled trial, Behav. Res. Ther. 45 (2007) 1085–1094. https://doi.org/10.1016/j.brat.2006.08.019.

[8] R. Moss-Morris, C. Sharon, R. Tobin, J.C. Baldi, A randomized controlled graded exercise trial for chronic fatigue syndrome: Outcomes and mechanisms of change, J. Health Psychol. 10 (2005) 245–259. https://doi.org/10.1177/1359105305049774.

[9] A. Deale, T. Chalder, S. Wessely, Illness beliefs and treatment outcome in chronic fatigue syndrome, J. Psychosom. Res. 45 (1998) 77–83. https://doi.org/10.1016/S0022-3999(98)00021-X.

[1] J.F. Wiborg, H. Knoop, M. Stulemeijer, J.B. Prins, G. Bleijenberg, How does cognitive behaviour therapy reduce fatigue in patients with chronic fatigue syndrome? the role of physical activity, Psychol. Med. 40 (2010) 1281–1287. https://doi.org/10.1017/S0033291709992212.

[2] J.M. Malouff, E.B. Thorsteinsson, S.E. Rooke, N. Bhullar, N.S. Schutte, Efficacy of cognitive behavioral therapy for chronic fatigue syndrome: A meta-analysis, Clin. Psychol. Rev. 28 (2008) 736–745. https://doi.org/10.1016/j.cpr.2007.10.004.

[3] J.R. Price, E. Mitchell, E. Tidy, V. Hunot, Cognitive behaviour therapy for chronic fatigue syndrome in adults, Cochrane Database Syst. Rev. 2008 (2008). https://doi.org/10.1002/14651858.CD001027.pub2.

[4] A.J. Wearden, R.K. Morriss, R. Mullis, P.L. Strickland, D.J. Pearson, L. Appleby, I.T. Campbell, J.A. Morris, Randomised, double-blind, placebo-controlled treatment trial of fluoxetine and graded exercise for chronic fatigue syndrome, Br. J. Psychiatry. 172 (1998) 485–492.

https://doi.org/10.1192/bjp.172.6.485.

[5] M. Sharpe, K. Hawton, S. Simkin, C. Surawy, A. Hackmann, I. Klimes, T. Peto, D. Warrell, S. Seagroatt, A. Nanke, Cognitive behaviour therapy for the chronic fatigue syndrome: A randomised controlled trial, Verhaltenstherapie. 8 (1998) 118–124. https://doi.org/10.1159/000030637.

[6] Cognitive Behavioural Therapy for Chronic Fatigue Syndrome: A Guide fo, (n.d.). https://www.routledge.com/Cognitive-Behavioural-Therapy-for-Chronic-Fatigue-Syndrome-A-Guide-for/Kinsella/p/book/9780415436120 .

[7] L. Quarmby, K.A. Rimes, A. Deale, S. Wessely, T. Chalder, Cognitive-behaviour therapy for chronic fatigue syndrome: Comparison of outcomes within and outside the confines of a randomised controlled trial, Behav. Res. Ther. 45 (2007) 1085–1094. https://doi.org/10.1016/j.brat.2006.08.019.

[8] R. Moss-Morris, C. Sharon, R. Tobin, J.C. Baldi, A randomized controlled graded exercise trial for chronic fatigue syndrome: Outcomes and mechanisms of change, J. Health Psychol. 10 (2005) 245–259. https://doi.org/10.1177/1359105305049774.

[9] A. Deale, T. Chalder, S. Wessely, Illness beliefs and treatment outcome in chronic fatigue syndrome, J. Psychosom. Res. 45 (1998) 77–83. https://doi.org/10.1016/S0022-3999(98)00021-X.
