# Supplementary material for: The added value of cognition-targeted exercise versus symptom-targeted exercise for multiple sclerosis fatigue: A randomized controlled pilot trial
Source: PLoS One. 2021 Nov 8;16(11):e0258752. doi: 10.1371/journal.pone.0258752 (PMC8575272; doi:10.1371/journal.pone.0258752)
Supplement: Attachment — Submitted filename: renamed_d18a7.docx [file pone.0258752.s004.docx]

**COMMENTS – Manuscript PONE-D-21-18476**

Title: “The Added Value of Cognition-targeted Exercise Versus Symptom-targeted Exercise for Multiple Sclerosis Fatigue: Randomized Controlled Trial”

**Important note: This review pertains only to ‘statistical aspects’ of the study and so** **‘clinical aspects’ [like medical importance,** **relevance of the study,** **‘clinical significance and implication(s)’ of the whole study, etc.] are to be evaluated [should be assessed] separately/independently. Further please note that any ‘statistical review’ is generally done under the assumption that (such) study specific methodological [as well as execution] issues are perfectly taken care of by the investigator(s). This review is not an exception to that and so does not cover clinical aspects {however, seldom comments are made only if those issues are intimately / scientifically related & intermingle with ‘statistical aspects’ of the study}. Agreed that ‘statistical methods’ are used as just tools here, however, they are vital part of methodology [and so should be given due importance].**

**COMMENTS:** The effect size is taken from a pilot study with total n=10 (i.e., 5 subjects in each group assuming 1:1 allocation ratio, which is too small to yield any useful/meaningful statistic) [Sample size section (line 163-7): The effect size estimate regarding the primary outcome MFIS, was obtained from a pilot study in which 10 participants underwent a similar protocol. The effect size for the differences between the groups was estimated to be 0.79. Accordingly, given a significance level of 5% and statistical power of 80%, 25 patients for each treatment arm were needed. To compensate for potential attrition, we increased the sample size by 20%.giving a total number of 30 patients per group]. The effect size used to estimate required sample size seems to be very large {unreasonably large}. In such (psychological therapy) studies [The objective of this study was to  explore the added value effect of cognition-targeted exercise versus symptom-targeted exercise for MS fatigue], the effect size achieved/attained/seen is generally (in most cases) is of small to ‘medium’ level.

By referring to table-2 on page 158 of J. Cohen’s paper “A power primer” in Psychological Bulletin, 1992, vol.:112, pp 155-159 even for medium effect size you need n=64 per group (type-I error=0.05, power=80%).

{Please note that the ‘effect size’ assumed should have some valid basis (exact published reference needs to be quoted) &/or reasonable/realistic, else the study is very likely ‘not to be able to’ detect a difference despite its presence}

Note that ‘The general linear model with repeated measures’ is not a technique originally developed (for testing interaction terms it is definitely nice, however, not) for between groups comparison. Direct between groups comparison techniques are available. Remember that though the measures/tools used are appropriate [e.g. modified fatigue impact scale (MFIS), work and social adjustment scale, hospital anxiety and depression scale, perceived stress scale] most of them yield data that are in [at the most] ‘ordinal’ level of measurement [and not in ratio level of measurement for sure {as the score two times higher does not indicate presence of that parameter/phenomenon as double (for example, a Visual Analogue Scales VAS score or say ‘depression’ score)}]. Then application of suitable non-parametric test(s) is/are indicated/advisable [even if distribution may be ‘Gaussian’ (i.e. normal)]. Agreed that there is/are no non-parametric test(s)/technique(s) available to be used as alternative in all situation(s) [suitable / most desired/applicable], but should be used whenever/wherever they are available. [Assumptions of ‘general linear model with repeated measures’ are unlikely to be fulfilled making their use invalid]

What is in lines 174-5 (we examined the results using 2 way repeated measures anova) is not reflected properly in tables [2 & 3]. Why there are two ‘P’ values [one each in column 4 & 5, row 4]. Application of repeated measures ANOVA yields only one ‘P’ (for each parameter/variable), in my knowledge. Then which test it is? If it is done after getting a significant ‘F’ by repeated measures ANOVA, then is not that “multiple testing” which requires adjustment in ‘P’? What the CI [in column 4 & 5, row 4] is for? Is it for difference in means? What is the interpretation? Moreover, one should use ‘to’ in/while presenting CI {example, [-5.9 -3.6] should be [-5.9 to -3.6]}.

It is well-known that while reporting [findings from] ‘Clinical Trial’ follow CONSORT guidelines. Even word ‘CONSORT’ or important items {like Random Sequence generation (Item 8a), Allocation concealment (Item 9)} of/in CONSORT checklist are not found [since your article type is ‘Clinical Trial’, you are supposed to cover these items in the report].

Two limitations of this study pointed out in lines 254-7 are not (unfortunately) the only ones. As pointed out in ‘important note’ above “This review pertains only to ‘statistical aspects’ of the study and so ‘clinical aspects’ should be assessed separately/independently. In my opinion, to rescue this article (which is quite possible), lot of re-vision is needed. Therefore, I have to unfortunately recommend ‘major revision’.
