# Supplementary material for: The added value of cognition-targeted exercise versus symptom-targeted exercise for multiple sclerosis fatigue: A randomized controlled pilot trial
Source: PLoS One. 2021 Nov 8;16(11):e0258752. doi: 10.1371/journal.pone.0258752 (PMC8575272; doi:10.1371/journal.pone.0258752)
Supplement: Attachment — Submitted filename: Response to Reviewers (1).docx [file pone.0258752.s005.docx]

Response to Reviewers

| Comments | Responses |
| --- | --- |
| Please ensure that your manuscript meets PLOS ONE's style requirements, including those for file naming. The PLOS ONE style templates can be found at https://journals.plos.org/plosone/s/file?id=wjVg/PLOSOne_formatting_sample_main_body.pdf and [https://journals.plos.org/plosone/s/file?id=ba62/PLOSOne_formatting_sample_title_authors_affiliations.pdf](about:blank) | Done as suggested |
| We note that you have indicated that data from this study are available upon request. PLOS only allows data to be available upon request if there are legal or ethical restrictions on sharing data publicly. For information on unacceptable data access restrictions, please see http://journals.plos.org/plosone/s/data-availability#loc-unacceptable-data-access-restrictions.  In your revised cover letter, please address the following prompts:  a) If there are ethical or legal restrictions on sharing a de-identified data set, please explain them in detail (e.g., data contain potentially identifying or sensitive patient information) and who has imposed them (e.g., an ethics committee). Please also provide contact information for a data access committee, ethics committee, or other institutional body to which data requests may be sent.  b) If there are no restrictions, please upload the minimal anonymized data set necessary to replicate your study findings as either Supporting Information files or to a stable, public repository and provide us with the relevant URLs, DOIs, or accession numbers. Please see http://www.bmj.com/content/340/bmj.c181.long for guidelines on how to de-identify and prepare clinical data for publication. For a list of acceptable repositories, please see http://journals.plos.org/plosone/s/data-availability#loc-recommended-repositories. | There is now restriction and we have uploaded the data file |
| Please amend your manuscript to include your abstract after the title page. | Done s suggested |
| Reviewer #1: Important note: This review pertains only to ‘statistical aspects’ of the study and so ‘clinical aspects’ [like medical importance, relevance of the study, ‘clinical significance and implication(s)’ of the whole study, etc.] are to be evaluated [should be assessed] separately/independently. Further please note that any ‘statistical review’ is generally done under the assumption that (such) study specific methodological [as well as execution] issues are perfectly taken care of by the investigator(s). This review is not an exception to that and so does not cover clinical aspects {however, seldom comments are made only if those issues are intimately / scientifically related & intermingle with ‘statistical aspects’ of the study}. Agreed that ‘statistical methods’ are used as just tools here, however, they are vital part of methodology [and so should be given due importance].  COMMENTS: The effect size is taken from a pilot study with total n=10 (i.e., 5 subjects in each group assuming 1:1 allocation ratio, which is too small to yield any useful/meaningful statistic) [Sample size section (line 163-7): The effect size estimate regarding the primary outcome MFIS, was obtained from a pilot study in which 10 participants underwent a similar protocol. The effect size for the differences between the groups was estimated to be 0.79. Accordingly, given a significance level of 5% and statistical power of 80%, 25 patients for each treatment arm were needed. To compensate for potential attrition, we increased the sample size by 20%.giving a total number of 30 patients per group]. The effect size used to estimate required sample size seems to be very large {unreasonably large}. In such (psychological therapy) studies [The objective of this study was to explore the added value effect of cognition-targeted exercise versus symptom-targeted exercise for MS fatigue], the effect size achieved/attained/seen is generally (in most cases) is of small to ‘medium’ level.  By referring to table-2 on page 158 of J. Cohen’s paper “A power primer” in Psychological Bulletin, 1992, vol.:112, pp 155-159 even for medium effect size you need n=64 per group (type-I error=0.05, power=80%). {Please note that the ‘effect size’ assumed should have some valid basis (exact published reference needs to be quoted) &/or reasonable/realistic, else the study is very likely ‘not to be able to’ detect a difference despite its presence} | I totally agree that this pilot small sample may be not representative for actual differences and may leads to a lack of representativeness in the study sample  And accordingly we changed our design to Randomized pilot trial  And recalculate the sample size based on the published minimal clinical difference for the primary outcome |
| Note that ‘The general linear model with repeated measures’ is not a technique originally developed (for testing interaction terms it is definitely nice, however, not) for between groups comparison. Direct between groups comparison techniques are available. Remember that though the measures/tools used are appropriate [e.g. modified fatigue impact scale (MFIS), work and social adjustment scale, hospital anxiety and depression scale, perceived stress scale] most of them yield data that are in [at the most] ‘ordinal’ level of measurement [and not in ratio level of measurement for sure {as the score two times higher does not indicate presence of that parameter/phenomenon as double (for example, a Visual Analogue Scales VAS score or say ‘depression’ score)}]. Then application of suitable non-parametric test(s) is/are indicated/advisable [even if distribution may be ‘Gaussian’ (i.e. normal)]. Agreed that there is/are no non-parametric test(s)/technique(s) available to be used as alternative in all situation(s) [suitable / most desired/applicable], but should be used whenever/wherever they are available. [Assumptions of ‘general linear model with repeated measures’ are unlikely to be fulfilled making their use invalid] | To follow up and compare the effects of the 2 alternative treatments over 3 months, we examined the results using a generalized Estimation Equation (GEE) Model .  Page 9 |
| What is in lines 174-5 (we examined the results using 2 way repeated measures anova) is not reflected properly in tables [2 & 3]. Why there are two ‘P’ values [one each in column 4 & 5, row 4]. Application of repeated measures ANOVA yields only one ‘P’ (for each parameter/variable), in my knowledge. Then which test it is? If it is done after getting a significant ‘F’ by repeated measures ANOVA, then is not that “multiple testing” which requires adjustment in ‘P’? What the CI [in column 4 & 5, row 4] is for? Is it for difference in means? What is the interpretation? Moreover, one should use ‘to’ in/while presenting CI {example, [-5.9 -3.6] should be [-5.9 to -3.6]}. | This part was rewritten again |
| It is well-known that while reporting [findings from] ‘Clinical Trial’ follow CONSORT guidelines. Even word ‘CONSORT’ or important items {like Random Sequence generation (Item 8a), Allocation concealment (Item 9)} of/in CONSORT checklist are not found [since your article type is ‘Clinical Trial’, you are supposed to cover these items in the report]. | We added the required information in Page 5 |
| Two limitations of this study pointed out in lines 254-7 are not (unfortunately) the only ones. | We added more relevant limitations  Page 13 |
| Reviewer #2: Title What did you need in the title Discuss What do you mean here by added value? The CBT used too much and published before, what was the new in your study What is your question and answer here? | I explained the rationale behind this study in the introduction part |
| Abstract: The background needs to be shortened. Methods section is poorly framed. It has to be re-written. Demographic profile of patients is not mentioned. P value are all .0005 – please check again poorly Key words and not illustrative (type of technique or therapy you used ) | Done as suggested |
| Introduction: 1. Explain the rationale of the study. Please delete information unrelated to objective so that the section is short and sweet. Kindly focus on three elements of introduction. a. What is known about the topic? (Background) b. What is not known? (The research problem) c. Why the study was done? (Justification) 2. Objective is not clear as mentioned above. Write on patients with multiple sclerosis cognitive behavioral model of MS fatigue Symptom-targeted Exercise objective of the current study | Done as suggested |
| Methods Totally unclear Rewrite by these consequences -Study design, setting, sample size, Participant (inclusion and exclusion criteria), -Which stage of MS you select –mention if the patients have any symptoms or remission –the patient under treatment during the study (mention all with its side effect ) -Intervention (explain CBT) - Comparison. Ethics and end point | I rewrote this part following these suggested arrangement and added more details for CBT  Medications at enrolment and types of MS were summarized at table 2 |
| LINE 101-102 psychological disorders or any chronic illness that may affect their fatigue discus what are you mean here | It is a typo error  I meant her psychiatric disorders.  Page 5 line 120  especially  Previous cross-sectional studies have demonstrated a close association between chronic fatigue and psychiatric disorders.  Skapinakis P, Lewis G, Meltzer H. Clarifying the relationship between unexplained chronic fatigue and psychiatric morbidity: results from a community survey in Great Britain. Am J Psychiatry. 2000;157:1492–1498. [[PubMed](https://www.ncbi.nlm.nih.gov/pubmed/10964867)]  Lawrie SM, Pelosi AJ. Chronic fatigue syndrome in the community. Prevalence and associations. Br J Psychiatry. 1995;166:793–797 |
| LINE 103 A third person blinded to group allocation Where were the 1st and 2nd person | The term "**third person**" refers to someone else, i.e., not the treatment provider or assessor .  And anyway I have changed to an independent person page 5  Line 123  To avoid any confusion |
|  |  |
| Line 110- 111 (explain CBT ) The subjects in the experimental group received eight 50-minute sessions of weekly CBT based on van Kessel’s model | We added more details for CBT table 1 |
| CONSORT 2010 checklist need revision pages not accurate for each element 13a and 13b not covered in page 8 Page 8 include (data analysis and results ) | Has been Revised |
| Results P value are all P<0.0005 in the 1st paragraph page 9 and 0.001 in the tables clarify please (why same results for all values ) Also in table 1 gender (numbers not clear )30-25 v 29-26 I think statistics need readjustment completely | We rewrote this part as suggested |
| Discussion: The discussion section needs to be written and described scientifically after correction needed for results | Done as suggested |
| References Rewrite again Most reference are old like (5,8,13,14,18,20,30) and not completed (volumes and issues ) like (33,34,35,29,26,15,1) | Done as suggested |
| Reviewer #3: This work explores a novel target for the treatment of fatigue in patients with MS, and I congratulate the authors for their efforts to carry out this trial. As additional data, it would be interesting to know the proportion of patients with different MS phenotypes recruited in the study, and if there were differences in the response to therapy in the different clinical forms. In addition, it would be interesting to know if the patients who intervened were under immunomodulatory treatment, and what type of treatment, since we know that interferons can cause fatigue Finally, an English correction by a native speaker would be recommended. | Done as suggested |
