# Supplementary material for: The added value of cognition-targeted exercise versus symptom-targeted exercise for multiple sclerosis fatigue: A randomized controlled pilot trial
Source: PLoS One. 2021 Nov 8;16(11):e0258752. doi: 10.1371/journal.pone.0258752 (PMC8575272; doi:10.1371/journal.pone.0258752)
Supplement: Attachment — Submitted filename: Response to Reviewers (1).docx [file pone.0258752.s006.docx]

Response to Reviewers

| Comments | Responses |
| --- | --- |
| Note that ‘The general linear model with repeated measures’ is not a technique originally developed (for testing interaction terms it is definitely nice, however, not) for between groups comparison. Direct between groups comparison techniques are available. Remember that though the measures/tools used are appropriate [e.g. modified fatigue impact scale (MFIS), work and social adjustment scale, hospital anxiety and depression scale, perceived stress scale] most of them yield data that are in [at the most] ‘ordinal’ level of measurement [and not in ratio level of measurement for sure {as the score two times higher does not indicate presence of that parameter/phenomenon as double (for example, a Visual Analogue Scales VAS score or say ‘depression’ score)}]. Then application of suitable non-parametric test(s) is/are indicated/advisable [even if distribution may be ‘Gaussian’ (i.e. normal)]. Agreed that there is/are no non-parametric test(s)/technique(s) available to be used as alternative in all situation(s) [suitable / most desired/applicable], but should be used whenever/wherever they are available. [Assumptions of ‘general linear model with repeated measures’ are unlikely to be fulfilled making their use invalid] | At the beginning, I would like to make it clear that I completely agree with the reviewer and that this Generalized estimating equations (GEE) was chosen after consulting a biostatistician . This biostatistician was added to the manuscript based on the required modifications  In this study, We have selected the Generalized estimating equations (GEE) ,which considered as an extension of generalized linear models (GLM) because the major strength of GEE is that they do not require the correct specification of the multivariate distribution  Especially the reviewer 1 has highlighted that a Generalized Linear Mixed Model (GLMM)require some parametric assumptions. And according to literature the Generalized estimating equations (GEE) are a nonparametric way to handle this issue (1)   (1) A Ziegler, M Vens -Generalized estimating equations . Methods of information in medicine, 2010 - thieme-connect.com   And accordingly, we have changed many sections in the manuscript  **Data analysis Page 9, line 109-201**  **Result, page 10, line 220-234**  **Abstract, line 43-44** |
| What is in lines 174-5 (we examined the results using 2 way repeated measures anova) is not reflected properly in tables [2 & 3]. Why there are two ‘P’ values [one each in column 4 & 5, row 4]. Application of repeated measures ANOVA yields only one ‘P’ (for each parameter/variable), in my knowledge. Then which test it is? If it is done after getting a significant ‘F’ by repeated measures ANOVA, then is not that “multiple testing” which requires adjustment in ‘P’? What the CI [in column 4 & 5, row 4] is for? Is it for difference in means? What is the interpretation? Moreover, one should use ‘to’ in/while presenting CI {example, [-5.9 -3.6] should be [-5.9 to -3.6]}. | We have changed the statistical technique used in this study  Instead of GLM we used Generalized estimating equations (GEE)  and accordingly, we have changed the tables completely  Now I believe the analysis technique is properly reflected in the table |
| It is well-known that while reporting [findings from] ‘Clinical Trial’ follow CONSORT guidelines. Even word ‘CONSORT’ or important items {like Random Sequence generation (Item 8a), Allocation concealment (Item 9)} of/in CONSORT checklist are not found [since your article type is ‘Clinical Trial’, you are supposed to cover these items in the report]. | We added the required information in Page 5 |
| Two limitations of this study pointed out in lines 254-7 are not (unfortunately) the only ones. | We added more relevant limitations  Page 13&14 lines 282 -289  we propose several limitations of this study, pointing to necessary future research work on this topic. First, our project only included a short-term follow-up of 3-months after intervention was terminated. It is unclear how long the identified fatigue related changes would remain and whether patient compliance may also be an issue. Second, because we chose participants with a scoring of less than six in the expanded disability status scale to investigate, it is unknown how our study results might relate to people with MS who have higher levels of disability. Third, within the resource constraints of a pilot investigation, only a small sample size was feasible. While our study had statistically significant findings and raised the possibility of Cognition-Targeted Exercise CTE benefits, no reliable conclusions about mood effects of CTE can be drawn from such a small sample. As Button et al [43] emphasize, “a study with low statistical power reduces the likelihood that a statistically significant result reflects a true effect.” Thus, replication studies with larger sample sizes are critical to further evaluating any potential mood effects of this type of exercise . Despite of the above mentioned limitations, the present study can be regarded as a positive step toward finding and adopting non-pharmacological interventions to ameliorate fatigue levels in patients with Multiple Sclerosis. |
